# Young age increases the risk for lymph node metastasis in patients with early Colon Cancer

**DOI:** 10.1186/s12885-019-5995-4

**Published:** 2019-08-14

**Authors:** Xin Xie, Jianhao Yin, Zhangjian Zhou, Chengxue Dang, Hao Zhang, Yong Zhang

**Affiliations:** Department of Surgical Oncology, The First Affiliated Hospital of Xi’an Jiaotong University, Xi’an, 710061 Shaanxi China

## Abstract

**Background:**

The risk of lymph node positivity in early-stage colon cancer is a parameter that impacts therapeutic recommendations. However, little is known about the effect of age on lymph node positivity in colon cancer with mucosal invasion. In this study, we aimed to quantify the effect of younger age on lymph node positivity in colon cancer with mucosal invasion.

**Methods:**

All patients were identified between 2004 and 2014 in the Surveillance, Epidemiology, and End Results database. Patients were stage T1-T2, did not undergo preoperative radiotherapy, had at least one lymph node examined, and underwent a standard colon cancer operation. Demographics and pathological data were compared between different age ranges. A nomogram model was built to estimate the probability of nodal involvement according to different characteristics. Decision curve analysis was performed by calculating the net benefits for a range of threshold probabilities.

**Results:**

This study identified 41,490 patients who met the eligibility criteria for our study. 1.4% (*n* = 620) of patients were under 40 years old; 5.9% (*n* = 2571) were between 40 and 49 years old. Within each T stage, positive lymph node rates decreased with increasing age. In univariate analyses, the positive lymph node rates for patients 20 to 39 years of age were significantly higher than in patients in the reference group for stages T1 and T2. After dividing the colon into the left and right parts, these trends remained. The lymph node metastatic rate was higher in the right colon than in the left colon in terms of different age ranges. The nomogram prediction system represents a novel model with which to estimate lymph node metastasis in early T stage colon adenocarcinomas based on four risk factors with a C-index of 0.657 (95% CI: 0.658–0666).

**Conclusions:**

Our study demonstrates that the risk of lymph node metastasis was higher in young (< 40 years) patients with early-stage colon adenocarcinomas. Therefore, more aggressive screening and therapeutic strategies should be considered for young patients with colon adenocarcinoma.

## Introduction

Colorectal cancer (CRC) is a commonly diagnosed malignancy that is estimated as the third most common cancer type in both males and females [1, 2]. The overall incidence and mortality of CRC have been reduced in recent decades, but the temporal patterns differ markedly by age [3]. Several studies have reported that the incidence and mortality of CRC has increased among adults under 50 years of age, whereas the death rate for adults older than 50 years has decreased by 34% from 2000 to 2014 [2–4]. Potential genetic predispositions, such as high levels of promoter methylation at CpG islands in CRC, as well as dietary habit changes, are considered potential risk factors for young CRC patients [2, 5–7]. Previous studies demonstrated that CRCs in younger patients are more likely to be located in the distal colon and rectum [3, 7]. Data from the proximal colon are limited.

The clinical outcomes of young adult patients with CRC remain controversial. Several studies demonstrated negative survival among young CRC patients compared to older patients [7–9], while recent studies have reported better outcomes among younger patients due to aggressive treatment strategies or the absence of frailty [3, 10, 11]. However, the survival benefits for young adult CRC patients might depend on early diagnosis [3, 12]. The aggressive features of CRC tumours in younger patients, including adverse histological grade, venous invasion and perineural invasion, were revealed in recent studies [6, 7, 13]. Current screening guidelines do not recommend routine screening for young adults, but for young adults with suspected CRC, several examinations are still necessary, such as colonoscopy and faecal occult blood test [3, 14].

.For young CRC patients, the assessment of lymph node status provides crucial information to guide treatment strategies. Numerous studies and guidelines have already recommended that examination of greater than 12 lymph nodes intraoperatively or postoperatively was minimum evaluative requirement for patients with CRC [15–17], and the involvement of an increased number of lymph nodes is associated with negative outcomes [18, 19]. A similar study in rectal carcinoma in younger patients indicated that an increased number of positive lymph nodes (LN+) was correlated with a young age at diagnosis [20]. To the best of our knowledge, the association of age at diagnosis and lymph node status has not been previously reported.

The Surveillance, Epidemiology, and End Results (SEER) database maintained by the National Cancer Institute is an open-access program available for incidence and survival analyses of cancer patients across the United States. In the current study, we analysed approximately 40,000 young patients with colon carcinoma in the most recent 10 years based on the SEER database and investigated the potential association between the status of lymph nodes examined and age at diagnosis.

## Methods

### Patients

Supported by National Cancer Institute, the SEER program collects demographic and clinicopathological information from local registries and covers approximately 28% of the United States population. Records of patients with colon cancer were obtained from the SEER database between 2004 and 2014. The inclusion criteria were as follows: 1. patients who were clinicopathologically diagnosed with adenocarcinoma of the colon; 2. patients who underwent surgery and for whom the exact pathological details were available; 3. patients with at least one lymph node resected. The exclusion criteria were as follows: 1.patients with distant metastasis; 2. patients who received radiotherapy prior to surgery (to eliminate the effect of preoperative radiation on lymph node harvest and positivity); 3. patients who underwent local excision or local destruction procedures (given the lack of expectations for obtaining lymph nodes with this type of procedure). In this retrospective study, a signed SEER research data agreement form was provided to the SEER program, and we were given approval to access and analyse the SEER data. There is no need for informed consent by analysing the SEER data. Besides, this study was also approved by the Ethics Committee of the First Affiliated Hospital of Xi’an Jiaotong University.

### Statistical analysis

All patients were regrouped according to the 8th American Joint Committee on Cancer TNM staging system. Predicting variables were evaluated for their association with lymph node involvement using univariate logistic regression models. A multivariate model was applied to all variables with a *P-*value less than 0.05. Associations were estimated using corresponding 95% confidence intervals (CIs). A nomogram was generated to estimate the probability of nodal involvement according to different characteristics. For the nomogram construction and validation, we randomly assigned two-thirds of the patients to the training set (*n* = 27,660) and one-third to the validation set (*n* = 13,830). The clinicopathological characteristics of the training and validation sets were evaluated. Harrell’s concordance index (C-index) was used to estimate the accuracy and identification abilities of the predictive factors. To estimate the clinical utility of the nomogram, decision curve analysis (DCA) was performed by calculating the net benefits for a range of threshold probabilities in the combined set of the training and validation cohorts. All statistical tests were two sided with 5% type I error. Statistical analyses were performed using R software version 3.3.2 (http://www.r-project.org) with the “SEERaBomb”, “rms” and “rmda” packages.

## Results

### Demographic and clinicopathological characteristics

We identified 41,490 patients who met the eligibility criteria for our study. Overall, 6358 patients (15.3% of the patient population) had at least one LN+. Within this cohort, approximately half were males (*n* = 20,292, 48.9%). The median age at diagnosis was 71 years (range,14 to 104 years old), with an average age of (mean ± SD)69.73 ± 12.43 years. Regarding the clinicopathological characteristics, adenocarcinoma invasion into the submucous layer was detected in 26,053 patients (62.8%). Most of the patients were Caucasian, 11.0% were black, and 7.5% were other ethnicities (including Chinese and Japanese descent). The clinicopathological details are presented in Table 1.

**Table 1:** The demographic and clinicopathological characteristics of patients

	Lymph nodes metastasis				*P* value
	Negative		Positive		
	Counts	Percentage	Counts	Percentage	
Gender					
Male	17,215	84.8%	3077	15.2%	
Female	17,917	84.5%	3281	15.5%	0.37
Age (year)					
≤ 40	414	69.9%	178	30.1%	
41–50	1913	77.9%	543	22.1%	
51–60	5470	81.1%	1273	18.9%	
61–70	8695	84.3%	1616	15.7%	
71–80	10,753	86.5%	1675	13.5%	
≥ 81	7887	88.0%	1073	12.0%	< 0.01^a^
Race					
White	28,841	85.4%	4950	14.6%	
Black	3747	81.8%	831	18.2%	
Others	2544	81.5%	577	18.5%	< 0.01
Size (mm)					
≤ 10	5426	89.0%	668	11.0%	
11–20	8180	85.5%	1390	14.5%	
21–30	8320	84.1%	1570	15.9%	
31–40	5913	83.2%	1190	16.8%	
41–50	3611	83.0%	742	17.0%	
≥ 51	3682	82.2%	798	17.8%	< 0.01
Mucinous					
Non-mucin	32,766	84.8%	5853	15.2%	
Mucin	2366	82.4%	505	17.6%	0.01
Grade					
Well	5782	90.5%	604	9.5%	
Moderate	26,388	85.0%	4667	15.0%	
Poor	2677	73.1%	985	26.9%	
Undifferentiated	285	73.6%	102	26.4%	< 0.01
Depth of invasion					
T1	13,694	88.7%	1743	11.3%	
T2	21,438	82.3%	4615	17.7%	< 0.01

^a^There were significant differences of the adjacent age groups

**Fig. 1:**
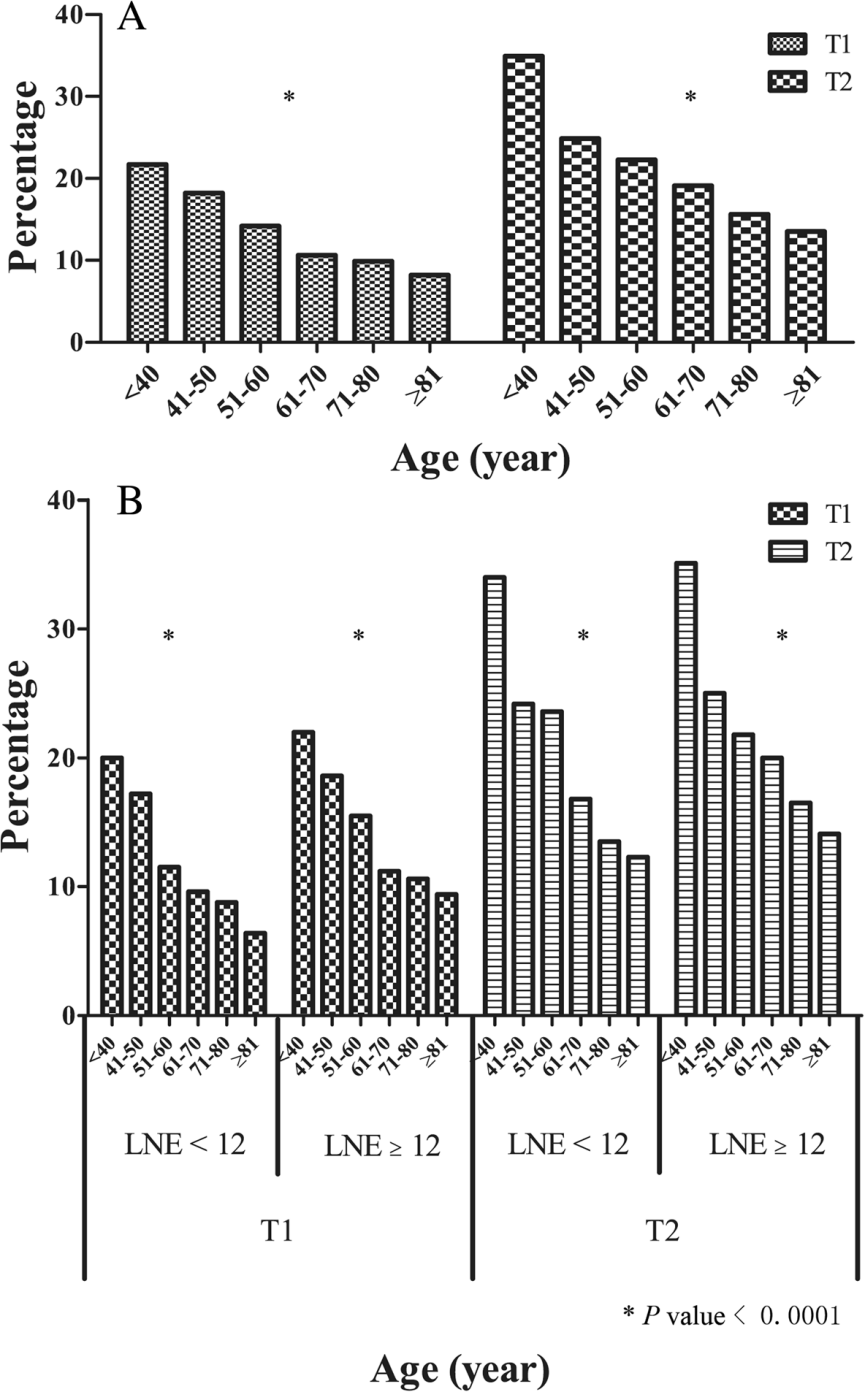
The distribution of lymph nodes positivity. **a**. Node positivity and age of diagnosis by depth of invasion. **b**. Node positivity and age of diagnosis by depth of invasion and number of lymph nodes examined. LNE = lymph nodes examined

Only 1.4% (*n* = 592) of patients were under 40 years of age; 5.9% (*n* = 2456) were between 40 and 49 years old, and 16.3% were between 50 and 59 years old. The median number of lymph nodes examined increased with T stage (median lymph nodes examined = 14 and 15 for T1 and T2 tumours, respectively). Within each T stage, the median number of lymph nodes examined decreased with increasing age. Regarding metastatic lymph nodes, the proportion of patients with at least one LN+ was 11.3and 17.7%forpatients with T1 and T2tumours, respectively. Within each T stage, the LN+ rates decreased with increasing age (within T stage, *P* < 0.001). The youngest patients had the highest LN+ rates within each T stage (Fig. 1a). In the univariate analyses, the LN+ rates for patients aged 20 to 39 years old were significantly greater than those in patients in the reference group for stages T1 and T2. Figure 1b presents the LN+ rate by age within T stage and further stratified by the number of examined lymph nodes (less than 12 and greater than 12examined lymph nodes). Within the T stage and examined lymph node group, an inverse association between age and metastatic lymph node rate remained statistically significant (*P* < 0.001). Furthermore, after the patients were divided into groups based on the affected side of the colon, these trends were maintained. Younger patients were more likely to exhibit lymph node metastasis. The lymph node metastasis rate was higher in patients with the right colon affected than in those with the left colon affected at different age ranges.

Nomogram prediction system for lymph node metastasis of early T stage colon adenocarcinoma.

**Fig. 2:**
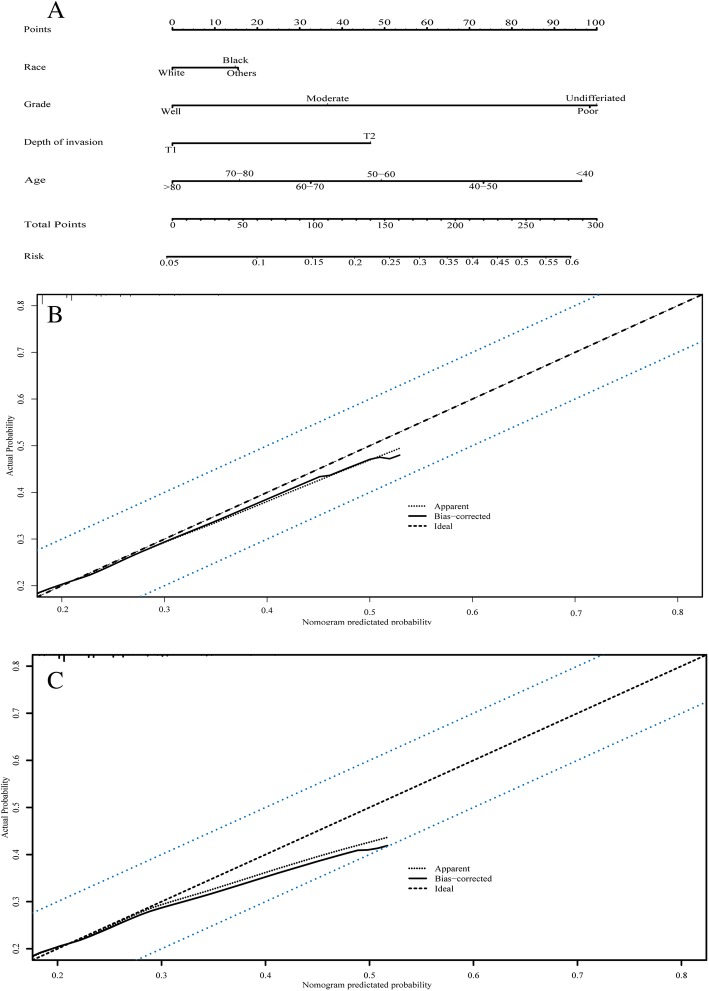
The predictive model of lymph nodes metastasis. **a**. Nomogram predicted lymph nodes metastasis risk using four available clinical characteristics. **b**. The calibration curve of the nomogram predicted system of the training set. **c**. The calibration curve of the validation set. The x-axis is the predicted lymph nodes metastatic risk calculated by the nomogram, and the y-axis is the actual metastatic status. The solid line represents the ideal reference line where predicted risk corresponds with the actual appearance, and the dotted lines represent a 10% margin of error. The actual status corresponded closely with the predicted lymph nodes metastasis and was always within the 10% margin of error

The nomogram prediction system represents a novel model with which to estimate the lymph node metastasis of early T stage colon adenocarcinomas based on four risk factors in the training set that exhibited significant differences in the multivariate analysis (Table 2): histological grade, depth of invasion, age at diagnosis and race. Each factor was ascribed a weighted point, and the total points indicated the risk of lymph node metastasis. For example, 39 years of age was associated with 95points, depth of invasion (T2) was associated with 47points, moderately differentiated adenocarcinoma was associated with 37points, and white ethnicity was associated with zero points, yielding a total score of 179 points. This score indicated that this patient had a 31% risk of regional lymph node metastasis. Local excision might not be sufficient. The factors and final nomogram model are presented in Fig. 2a. To evaluate the predictive accuracy of the nomogram prediction system, the C-index of the training set was calculated and validated. For the nomogram model built with the training set, the C-index was 0.633 (95% CI: 0.624–0.642). For the validation set, the C-index was 0.633 (95% CI: 0.620–0.646). The calibration curves of the training and validation sets are presented in Fig. 2b and c.

**Table 2:** The risk factors of predicted lymph node metastasis

	Lymph nodes metastasis		
	Univariate	Multivariate	
	P value	P value	Hazard Ratio
Gender	0.678		
Age (year)	< 0.001	< 0.001	0.976–0.982
Race	< 0.001	< 0.001	1.053–1.174
Size (mm)	< 0.001	0.053	1–1.003
Mucinous	0.016	0.085	0.985–1.267
Grade	< 0.001	< 0.001	1.612–1.827
Depth of invasion	< 0.001	< 0.001	1.045–1.061

**Fig. 3:**
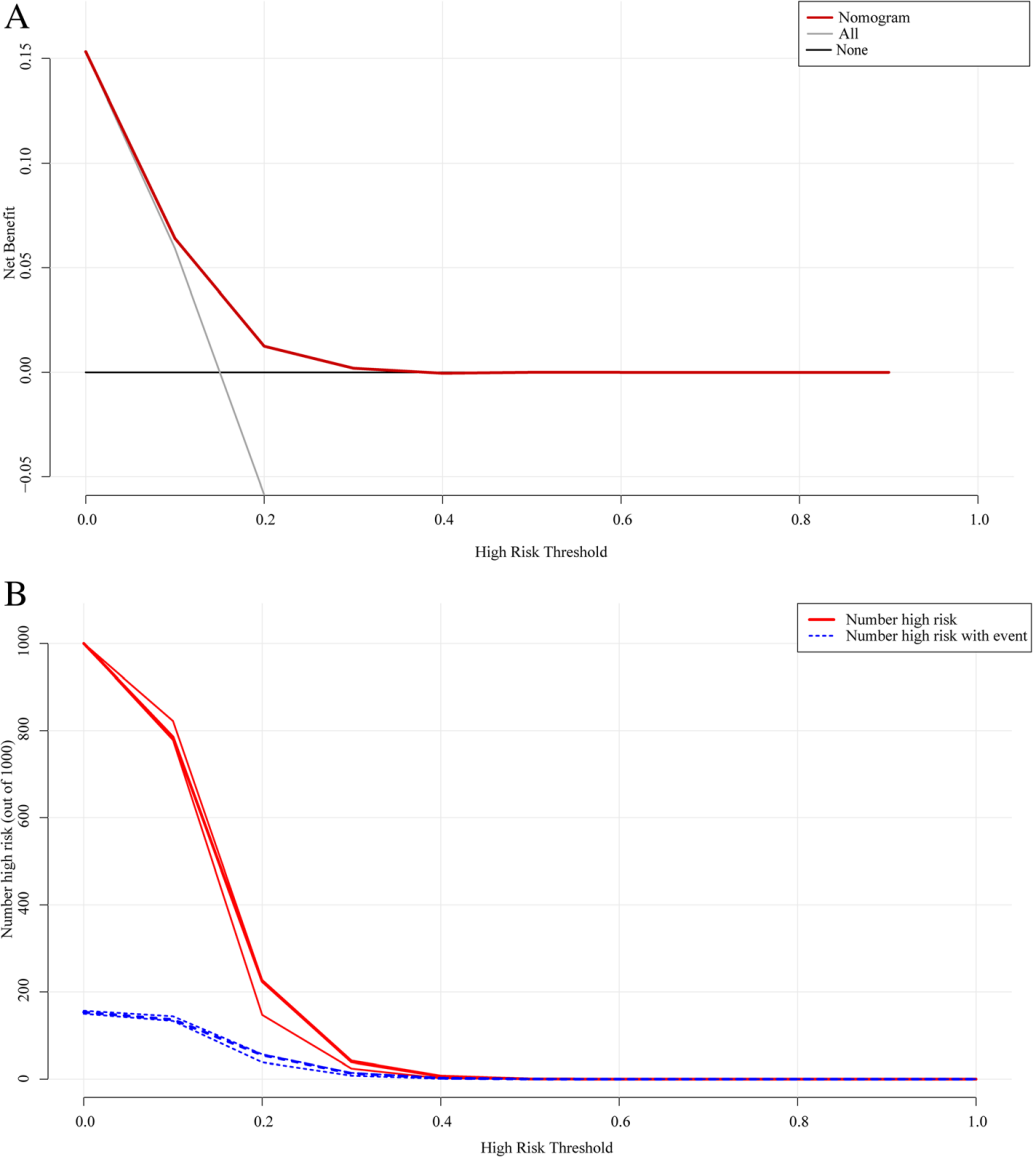
The decision curve analysis for the nomogram model. **a**. Decision curve analysis for the nomogram predictive model. The y-axis represents the net benefit. The red line represents the nomogram model. The grey line represents the hypothesis that all patients had lymph node metastases. The black line represents the hypothesis that no patients had lymph node metastases. The x-axis represents the threshold probability. The threshold probability is where the expected benefit of treatment is equal to the expected benefit of avoiding treatment. For example, if the possibility of lymph node metastasis involvement of a patient is over the threshold probability, then a treatment strategy for lymph node metastasis should be adopted. The decision curves in the validation set showed that if the threshold probability is between 0 and 0.4, then using the nomogram to predict lymph node metastases adds more benefit than treating either all or no patients. **b**. The cost-benefit ratio of the nomogram predictive model. The red curve represents the number of people classified as positive (high risk) at each threshold probability; the blue curve is the number of true positives for each threshold probability

The DCA for the nomogram is presented in Fig. 3. The DCA indicated that when the threshold probability is within the range of 0 to 0.4, the nomogram prediction model adds more net benefit than the “treat all (standard operation)” or “treat none (local excision)” strategies do.

## Discussion

Although the overall incidence and mortality of CRC has decreased recently, CRC in younger patients (< 40 years) has exhibited the opposite trend. You et al. reported that the annual percentage change for CRC in younger patients was 2.1% since 2001 vs. -2.5% for patients with late-onset CRC [8]. However, due to the limitations of the current CRC screening guidelines and low level of suspicious for potential clinical symptoms, diagnostic delays often occur in young adults with CRC [3, 14, 21]. Distinct from elder CRC patients, young-onset patients presents different clinicopathological characteristics: poorly differentiated tumors, left-sided location and rectal, which indicates more clinical considerations are needed for young CRC patients [21]. For young adults with CRC, local lymph node assessments are crucial for both therapeutic strategies and prognostic prediction [22, 23]. In the current study based on the nationwide SEER database, we found that lymph node metastasis was more common in younger patients with colon adenocarcinomas, especially in the early T stage (Fig. 1). In addition, we further investigated a novel nomogram prediction model that is convenient to clinically estimate the risk of lymph node metastasis in young patients with early-stage colon adenocarcinoma.

Several factors explain the increased incidence of young patients with CRC. The genetic and biological behaviours differ considerably between patients < 40 years and elderly patients. High levels of promoter methylation at CpG islands (CIMP-H) and microsatellite instability (MSI) were identified in CRC specimens [7, 24, 25]. As a critical molecular mechanism of CRCs, CIMP-H carcinomas exhibit abnormal Wnt/β-catenin pathway signalling and *KRAS* mutations, which represent an essential conventional adenocarcinoma sequence in CRCs [26, 27]. However, a recent single-centre study revealed that CIMP-H was not significantly important in young patients with CRCs [7]. Thus, the molecular aetiology and mechanisms of CRCs in young patients are still unclear and require further investigation.

Despite the potential genetic differences between young and elderly patients with CRC, tumours from young (age < 40) CRC patients tend to exhibit more aggressive behaviours and adverse histological grades. An analysis of SEER data from 1973 to 1999 demonstrated that young CRC patients (age < 40) presented with more advanced-stage cases and more distant metastases than patients with age > 40 years [13]. Chang et al. reported that young CRC patients presented with or developed metastatic disease more frequently than elderly CRC patients (45% vs. 25%, *P* = 0.014) [7]. In addition, young CRC patients were more likely to exhibit adverse histological grades or conditions associated with aggressive features, including regional lymph node metastasis, venous invasion, perineural invasion, mucinous features and signet ring cell carcinoma [6, 7, 12]. Consistently, a cohort of 330 patients with CRC presented different tumor characteristics in patients age ≤ 40 years, which indicates young patients with CRC exhibits distinct tumor clinicopathological profiles in comparison with elder patients [28]. Together with differences in molecular mechanism involved in young CRCs, age-specific assessment and therapeutic strategies should be considered for CRC patients who are younger than 40 years of age.

Lymph node assessment is critical to both CRC therapeutic strategies and prognostic prediction. Yantiss et al. reported more frequent lymphovascular (83% vs.51%, *P* = 0.03) invasion in young (< 40 years) CRC patients, which suggests more aggressive biological characteristic of young CRCs [6]. Several factors are significantly associated with lymph node metastasis in CRC patients, including T stage, histological grade and number of lymph nodes examined intraoperatively or post-surgically [29, 30]. Besides, tumor budding, firstly described by Imai in the 1950s, was recognized as an independent risk factor for CRC outcomes in recent few years [31, 32]. Especially in malignant polyps and Stage I/II of CRC, tumor budding was associated with increased risk of LN metastasis [32–35]. Overall survival can be improved by increasing the number of lymph nodes examined in CRC patients [3, 18–20]. Thus, the examination of a minimum of 12 lymph nodes was recommended for patients with CRC [17]. In our study, we found that the number of lymph nodes examined in patients with colon adenocarcinoma increased with T stage, which was consistent with the results of a previous study in rectal adenocarcinoma [20]. To the best of our knowledge, reports of the association between lymph node metastasis and T stage as influenced by age in colon adenocarcinoma are limited. In the current study, SEER data were stratified by different T stages. In each T stage, LN+ were significantly decreased with increasing age, and the LN+ rates of young (age 20 to 39 years) colon adenocarcinoma patients were increased compared with those in the early T stage (T1 and T2) reference group (Fig. 1). Similar results were reported in a recent study of rectal cancer by Meyer et al., which revealed that young patients with early-stage rectal cancer exhibited an increased risk of LN+ status^20^. In addition, given the different clinicopathological characteristics between left colon carcinomas (LCCs) and right colon carcinomas (RCCs) [36], lymph node status and age distributions were further investigated. We found that the association between age and LN+ rates still existed in both the LCC and RCC groups, which further supported our finding that early-stage colon adenocarcinomas in younger patients were more likely to exhibit lymph node metastasis.

To predict lymph node status more easily in the clinic, we generated a novel nomogram prediction model including four risk factors: histological grade, depth of invasion, age at diagnosis, and race (Fig. 2). High nomogram prediction scores indicate a high risk of LN+, and further consideration for therapeutic strategies on young CRC patients is needed. To evaluate the accuracy and clinical application potential, we introduced the C-index and DCA to analyse the training and validation sets [37, 38]. Our nomogram prediction model showed a close correspondence with the actual status of lymph nodes metastasis (Fig. 2b, c). However, as the C-index of nomogram prediction model was 0.633, larger cohort studies and further modification are needed and worth expectation. With benefits of directly application and no need for additional information collection, the DCA was widely used to evaluate the clinical prediction models [38, 39]. In the current study, nomogram prediction model exhibited the threshold of 0.4 in DCA method (Fig. 3), which meant the nomogram showed more net benefit for clinical prediction within high risk threshold of 0–0.4.Above all, our nomogram prediction model is a potentially useful tool to estimate lymph node status and prognosis in young patients with early-stage colon adenocarcinomas.

Given the complicated aetiological mechanism and aggressive biological behaviours of early-stage colon adenocarcinomas in young patients, more aggressive extend lymph nodes resection and multidisciplinary therapeutic strategies should be considered [28, 40, 41]. Sarli et al. demonstrates the number of examined lymph nodes decreased with increased CRC patient age [23]. Further, a cohort from Quan et al. indicates more lymph nodes were retrieved from surgical specimen than older patients [40]. Consistent with previous reports, in our study, young adult (age < 40 years) patients with early-stage colon adenocarcinomas shows high lymph node metastasis risks despite low T stages. Regional resections or endoscopic mucosal resections (EMRs) might not be sufficient, which could cause inadequate lymph nodes retrieval and increase the risk of local recurrence [12, 42, 43]. Although adjuvant chemotherapy provides significant survival benefit for high-risk Stage II (i.e. T4 tumors, high-grade histology, lymphovascular invasion and suboptimal margins) and Stage III patients with colon cancer [44–46], it’s still controversial on applying adjuvant chemotherapy for young patients with colon adenocarcinoma [46, 47], which indicates further investigation and clinical trials are still needed for young patients.

## Limitations

Several limitations of our study should be noted. First, given that the SEER database is a nationwide program, several diagnostic criteria, such as histological grades and differentiation between rectosigmoidal and rectal cancers, and verification of tumour locations might be subjective, which could cause potential systematic bias. In addition, the detailed pathological data were limited in the SEER database. Several risk factors, such as lymphovascular invasion and tumor budding, which were associated with LN metastasis were lacking and needed further assessment to consist with our nomogram model. Besides, the details of family history of CRC from young patients were limited, which may cause bias in selection and prognostic prediction. Finally, the Harrell’s C-index of our nomogram prediction system is 0.633, which indicates our model needs larger cohort data for further validation and modification.

## Conclusions

In conclusion, our study demonstrates that the risk of lymph node metastasis is increased in young (< 40 years) patients with early-stage colon adenocarcinomas. Therefore, more aggressive therapeutic strategies should be considered for young patients with colon adenocarcinomas. Especially for young patiens meet the criteria of EMR or ESD, which is relatively less invasive than surgery but could not detect enough regional lymph nodes, it might be not sufficient only apply EMR or ESD due to greater risk of LN+ than older patients with same T stage. We also generated a novel nomogram prediction model to assess lymph node metastasis. Given that the nomogram includes four potential risk factors, our nomogram model is accurate and convenient for clinical utilization.

## Data Availability

Colon cancer patient records were obtained from the National Cancer Institute’s SEER database.

## Abbreviations

CIMP-H: High levels of promoter methylation at CpG islands
C-index: Harrell’s concordance index
CRC: Colorectal cancer
DCA: Decision curve analysis
EMRs: Endoscopic mucosal resections
MSI: Microsatellite instability
SEER: Surveillance, Epidemiology, and End Results
